# Atrophic gastritis and chronic diarrhea due to *Helicobacter pylori* infection in early infancy: A case report

**DOI:** 10.1097/MD.0000000000017986

**Published:** 2019-11-22

**Authors:** Toshihiko Kakiuchi, Aiko Nakayama, Ryo Shimoda, Muneaki Matsuo

**Affiliations:** a Department of Pediatrics; b Internal Medicine, Faculty of Medicine, Saga University, Saga, Japan.

**Keywords:** atrophic gastritis, chronic diarrhea, eradication, *Helicobacter pylori*, infancy

## Abstract

**Rationale:**

*Helicobacter pylori* infection causes atrophic gastritis in childhood, but atrophic gastritis due to *H pylori* infection is extremely rare in infancy. The relationship between *H pylori* infection and chronic diarrhea without protein leakage remains controversial.

**Patient concerns:**

An 8-month-old male infant presented to our hospital with severe watery diarrhea, erythema, and failure to thrive from approximately 1 month after birth. Blood, stool, esophagogastroduodenoscopy, total colonoscopy, and *H pylori* urease analysis results were positive, thereby suggesting atrophic gastritis.

**Diagnoses:**

Atrophic gastritis and chronic diarrhea due to *H pylori* infection.

**Interventions:**

We performed *H pylori* eradication therapy using triple therapy with vonoprazan (6 mg/kg), amoxicillin (300 mg/d), and clarithromycin (120 mg/kg) for 7 days.

**Outcomes:**

From approximately 1 week after the *H pylori* eradication therapy, the frequency of defecation had decreased, stool shape had improved, and body weight had gradually increased.

**Lessons:**

*H pylori* infection can cause atrophic gastritis and chronic diarrhea even in infancy. Early eradication therapy for *H pylori* infection may be useful for prevention of gastric cancer and improvement in growth disorders.

## 1 Introduction

*Helicobacter pylori (H. pylori)* infection is generally established in the family at ≤5 years of age in Japanese children^[1]^ and is known to cause atrophic gastritis in childhood.^[2–5]^ Atrophic gastritis due to *H pylori* infection is extremely rare in infants; therefore, only few studies have reported this condition in infancy to early childhood.^[5]^*H pylori* infection is also known to be one of the causes of Ménétrier disease^[6,7]^ and protein-losing gastroenteropathy,^[8]^ which leads to protein leakage and diarrhea. There are a few studies that have reported the correlation between Ménétrier disease and *H pylori* infection, chronic diarrhea, and malnutrition.^[9]^ Conversely, *H pylori* infection has not been shown to cause diarrhea without protein leakage.^[10]^

Here we report a case of an infant with atrophic gastritis and chronic diarrhea due to *H pylori* infection who was successfully treated with eradication therapy, with recovered growth.

## 2 Case presentation

**Figure 1:**
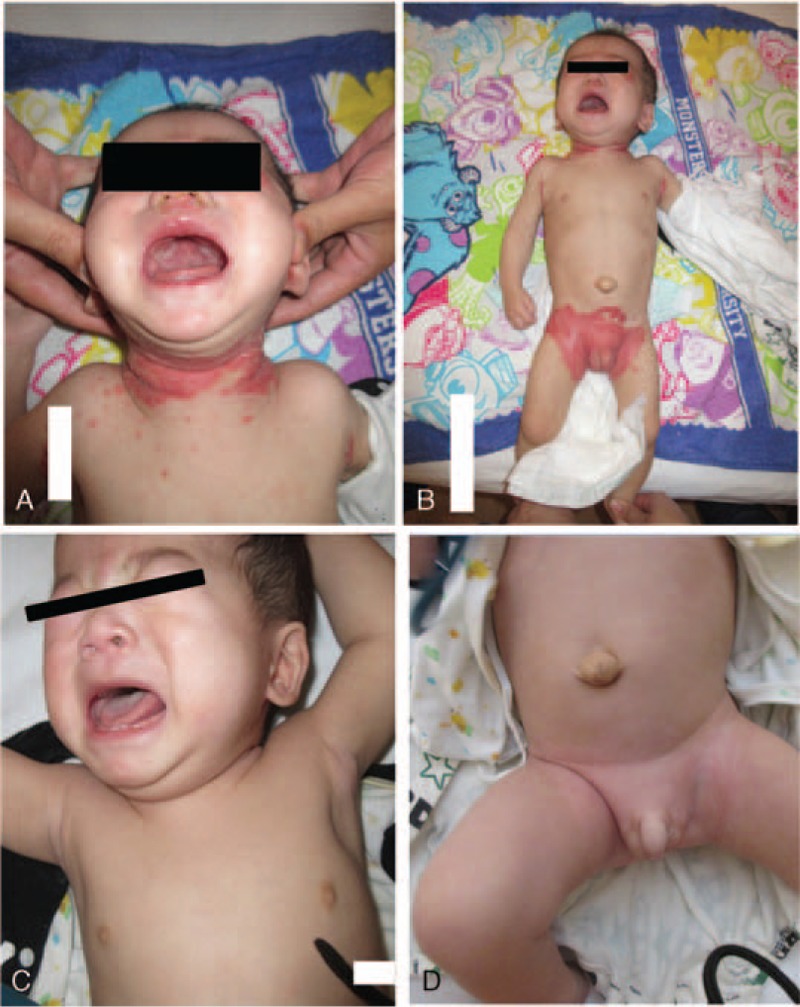
(A, B) Physical examination revealed erythema and crust around the opening area of mouth, eyelid rim, nostril, anal, and penis on admission. The trunk was barely thin. (C, D) After biotin administration, the erythema and crust disappeared quickly and the skin appeared normal.

An 8-month-old male infant presented to our hospital with severe watery diarrhea and erythema of the face, neck, and trunk (Fig. 1A and B). From approximately 1 month after birth, the patient had severe watery diarrhea and poor body weight gain. At 3 months of age, he was suspected of having a milk allergy and was hydrolyzed milk; however, his diarrhea did not improve and erythema gradually appeared. The results of the milk-specific immunoglobulin E, allergen-specific lymphocyte stimulation tests for casein, lactoferrin, and lactalbumin as well as the stool eosinophil test performed by the previous doctor were normal, but symptoms worsened and the patient was referred to our hospital for closer examination of his digestive tract.

**Figure 2:**
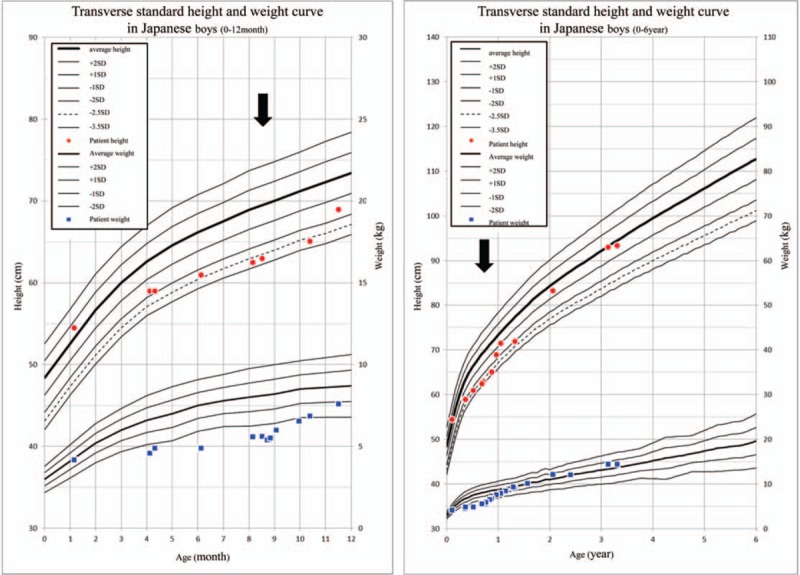
At admission, the height and body weight of the patient were below − 2.0 standard deviations. After *Helicobacter pylori* eradication therapy, his growth improved drastically in a short time. *H pylori* eradication therapy; *SD = *standard deviation Black arrow.

On admission, the patient had more than 8 episodes of diarrhea per day. His height was 63.0 cm (−3.1 standard deviation [SD]) and body weight was 5.6 kg (−3.0 SD), demonstrating a failure to thrive (Fig. 2). His vital signs were normal: body temperature was 37.4°C, heart rate was 115 beats/min, and blood pressure was 92/44 mm Hg. Physical examination revealed erythema and crust around the opening area of his mouth, eyelid rim, nostril, anus, and penis His hair was normal and slightly thin, and neurological development was generally normal. There were no obvious abnormal findings in the chest and abdomen except for an umbilical hernia.

Table 1 shows the blood test results. The patient's white blood cell count was 11,200/μL (normal range: 3300–8600/μL), eosinophil percentage was normal (<5.6%), and hemoglobin level was 11.2 g/dL (normal range >10.5 g/dL). His laboratory data were as follows: total protein level was 5.9 g/dL (normal range 4.3–7.3 g/dL), serum albumin was 3.8 g/dL (normal range 2.5–4.5 g/dL), total cholesterol was 106 mg/dL (normal range 130–220 mg/dL), triglyceride was 105 mg/dL (normal range 36–130 mg/dL), transthyretin was 14.2 mg/dL (normal range 22.0–40.0 mg/dL), transferrin was 258 mg/dL (normal range 240–400 mg/dL), retinol binding protein was 1.4 mg/dL (normal range 2.4–7.0 mg/dL), immunoglobulin G was 593 mg/dL (normal range >234 mg/dL), CD3^+^T was 65.4% (normal range 50%–77%), CD19^+^B was 19.3% (normal range 13%–35%); CD16^−^56^+^ was 13.8% (normal range 2%–14%), C3 was 91 mg/mL (normal range 65–135 mg/mL), C4 was 18 mg/dL (normal range 13–40 mg/dL), and CH50 was 60 U/mL (normal range 25–48 U/mL). The patient's liver function, kidney function, and electrolyte data were in the normal range. Serum and urine *H pylori* antibody tests were negative. Urinalysis and urine sediment test results were normal. The stool bacterial culture test did not detect any pathological bacteria, and eosinophils in feces were negative. The concentration of alpha-1 antitrypsin in stool was 10.9 mg/dL (normal range <33 mg/dL). The stool antigen test for *H pylori* was negative. Chest and abdomen radiography, head plain computed tomography (CT), and abdominal enhanced CT revealed no obvious abnormal findings.

**Table 1:**
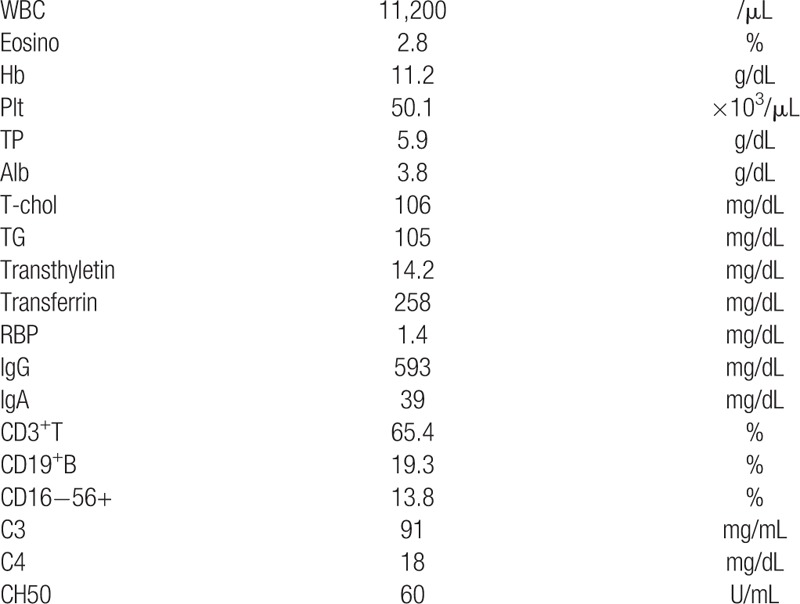
Blood test results at the time of admission.

**Figure 3:**
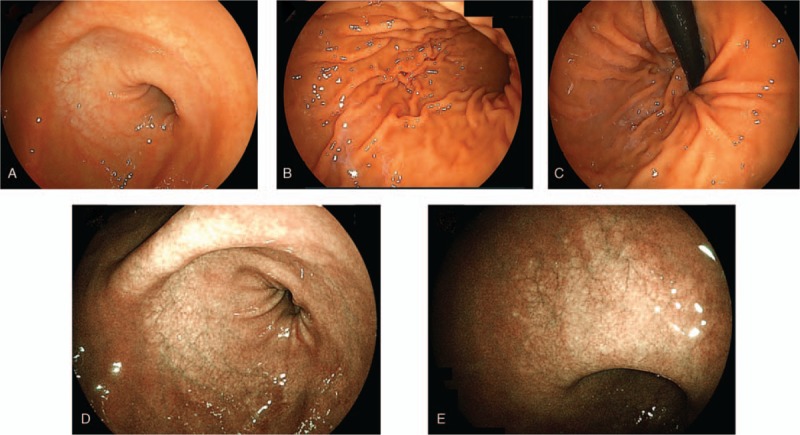
Esophagogastroduodenoscopy revealed gastric mucosal atrophy localized from the antrum to the upper corner. The atrophic grade was C-2 according to the Kimura–Takemoto classification system. The esophagus and duodenum did not present any abnormality. Linked color imaging findings for (A) antral zone, (B) gastric body, (C) cardia and fornix, (D) antral zone, (E) gastric angle.

**Figure 4:**
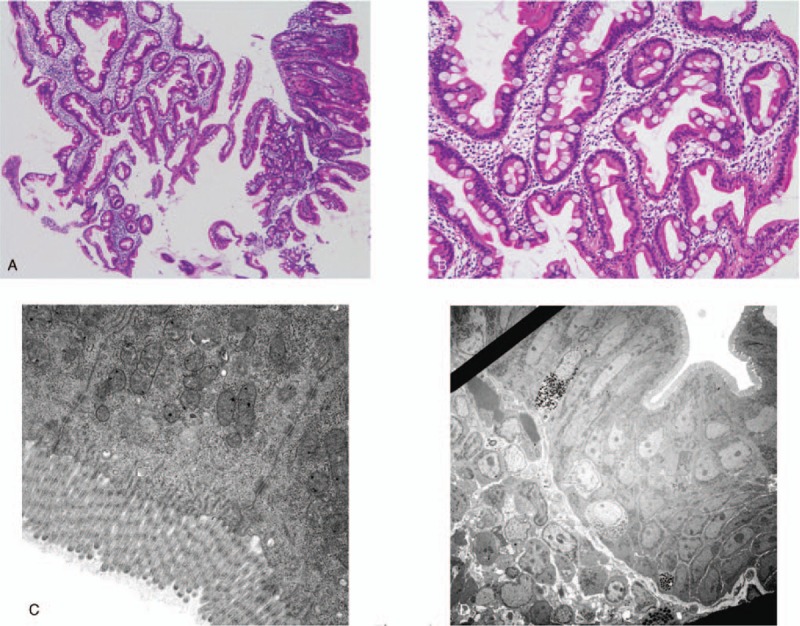
(A, B) The only pathological findings of the duodenal mucosa were mild edema and infiltration of inflammatory cells in the lamina propria (HE stain). (C, D) The electronic microscopic images showed no abnormal villi. *HE = *hematoxylin and eosin.

**Figure 5:**
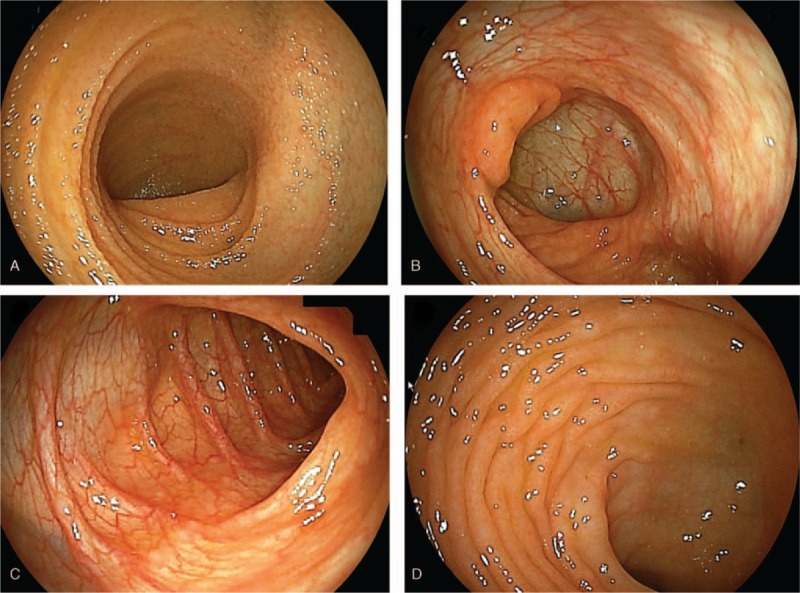
Total colonoscopy revealed no obvious localized lesions from the terminal ileum to the rectum. (A) terminal ileum, (B) ileocecum, (C) descending colon, (D) rectum.

**Figure 6:**
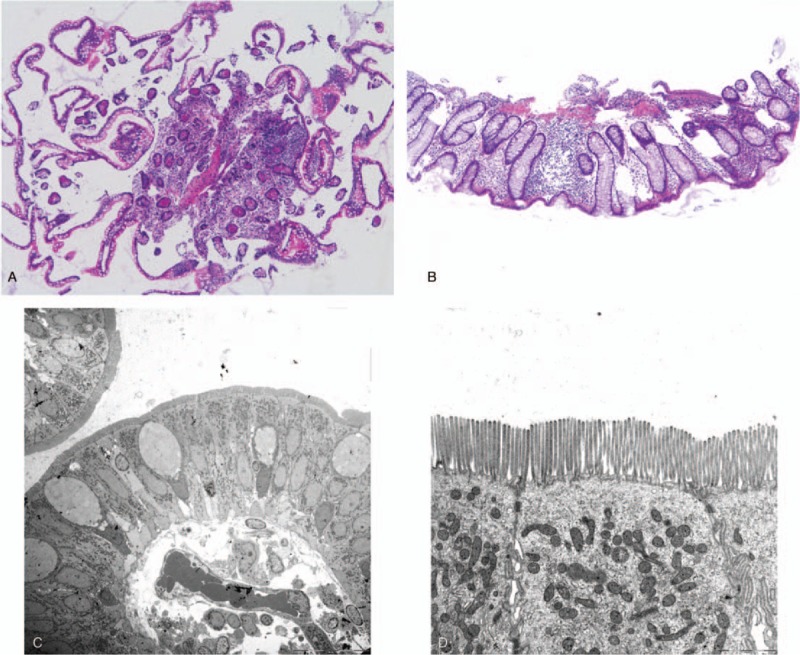
(A, B) The only pathological findings of the terminal ileum and rectum mucosa were mild edema and infiltration of inflammatory cells in the lamina propria (HE stain). (C, D) The electronic microscopic images showed no abnormal villi, similar to that for the duodenum. *HE = *hematoxylin and eosin.

To observe the presence of digestive tract diseases, we performed esophagogastroduodenoscopy (EGD) and total colonoscopy (TCS). Figure 3 presents the findings of EGD. No abnormal findings were found in the esophagus, but the stomach showed atrophic mucosa localized from the antrum to the upper corner. The degree of atrophy according to the Kimura–Takemoto classification system for atrophic gastritis was grade C-2.^[11]^ No obvious abnormal findings were observed from the duodenal bulb to the second portion. The only pathological findings of the duodenal mucosa were mild edema and infiltration of inflammatory cells in the lamina propria (Fig. 4A and B). Electronic microscopic images showed no abnormal villi (Fig. 4C and D). The rapid urease test using gastric mucosa was positive. TCS revealed no obvious localized lesions from the terminal ileum to the rectum (Fig. 5). The only pathological findings of the terminal ileum and rectum mucosa were mild edema and infiltration of inflammatory cells in the lamina propria (Fig. 6A and B). Electronic microscopic images showed no abnormal villi, similar to that for the duodenum (Fig. 6C and D).

There were no obvious abnormal findings for the causative agent of diarrhea and poor weight gain other than the positive *H pylori* urease test. Therefore, we performed *H pylori* eradication triple therapy using vonoprazan (6 mg/kg), amoxicillin (300 mg/d), and clarithromycin (120 mg/kg) for 7 days. There were no side effects during or after this eradication treatment. From approximately 1 week after *H pylori* eradication therapy, the frequency of defecation was decreased, stool shape was improved, and body weight was gradually increased. The only other treatment in addition to *H pylori* eradication therapy was that including coping treatments such as nutrition administration, hydration, vitamin supplementation, and probiotics. After treatment, the diarrhea had completely disappeared and the body weight had steadily increased, reaching the mean value for transverse standard height and weight curve in Japanese boys 1 year later. Skin erythema was rapidly improved by biotin administration because it was thought to be due to the long-term consumption of allergy milk containing no biotin (Fig. 1C and D).

**Figure 7:**
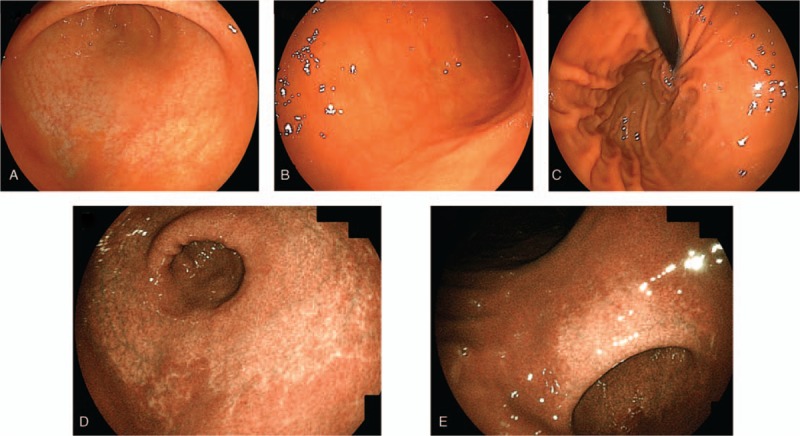
At 2 yr and 6 mo after *Helicobacter pylori* eradication therapy, esophagogastroduodenoscopy revealed that the degree of gastric mucosal atrophy was unchanged. Linked color imaging findings for (A) antral zone, (B) gastric body, (C) cardia and fornix, (D) antral zone, (E) gastric angle.

**Figure 8:**
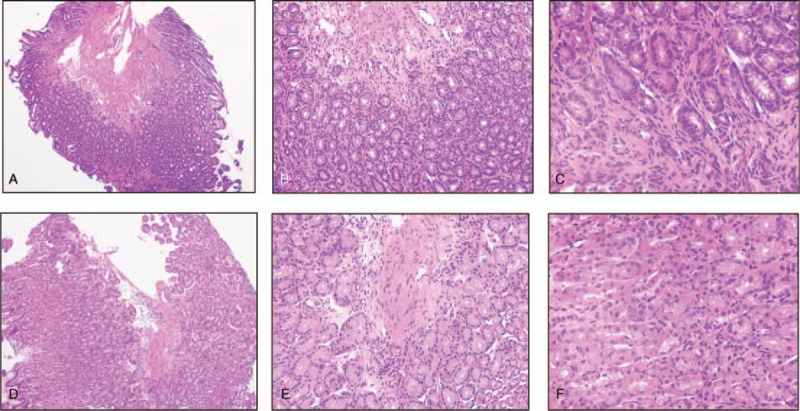
The pathological findings showed atrophy of the crypt epithelium and infiltration of chronic inflammatory cells into the stroma in both the antrum (A–C) and stomach body (D–F).

At 3 years and 3 months of age, EGD was performed for the purpose of follow-up (Fig. 7). Atrophic gastric mucous spreading from the entire vestibular area to the side of the comet was observed; it was rated as grade C-2 according to the Kimura–Takemoto classification system, which was the same as the previous test. Figure 8 presents the pathological findings. Atrophy of the crypt epithelium and infiltration of the chronic inflammatory cells into the stroma were noted in both the antrum (Fig. 8A–C) and stomach body (Fig. 8D–F). The pathological findings were suggestive of atrophic gastritis. The rapid urease test for *H pylori* was negative, serum pepsinogen I was 34.1 ng/mL, and pepsinogen I/II ratio was 4.2. At present, no diarrhea symptoms are observed, and height and body weight has remained within the normal range for Japanese boys

## 3 Discussion

The clinical course of the patient's disease provided 2 important clinical suggestions. First, *H pylori* infection can cause atrophic gastritis even in early infancy. Several studies have suggested that *H pylori* eradication in young children before gastric mucosa atrophy can effectively prevent new gastric cancers.^[12,13]^ This work lead to clinical trials for screening and treatment of *H pylori* infection among junior high school students as a prevention strategy against gastric cancer in Japan.^[14,15]^ However, *H pylori* eradication therapy for children at <15 years of age is not covered by insurance in Japan. Severe gastric mucosal atrophy and submucosal inflammation are rarely observed in children^[16–18]^; moreover, *H pylori* eradication therapy is not recommended in junior high school students because children have a low risk of differentiated gastric cancer.^[16,17]^ In our study, atrophic gastritis was recognized in early childhood and gastric mucosal atrophy persisted for >2 years after eradication. Because gastric mucosal atrophy is a cause of gastric cancer,^[19]^ we believe that *H pylori* eradication must be performed as soon as possible.

Second, *H pylori* infection can cause chronic diarrhea without protein leakage as well as growth failure. The patient in our study presented with chronic diarrhea with no apparent protein leakage in the stool. No abnormal findings were observed except the positive *H pylori* urease test; therefore, *H pylori* eradication therapy drastically improved the diarrhea. Although the relationship between *H pylori* infection and chronic diarrhea without protein leakage remains controversial, chronic diarrhea positive for *H pylori* infection should be considered an indication for eradication treatment. Although *H pylori* infection may play a protective role against bacterial diarrhea in children,^[20]^ it is known to affect the gastric and intestinal microbiota^[21–23]^ and may cause chronic diarrhea due to any cause other than infection. In our study, we found that long exposure to *H pylori* infection possibly decreased the growth rate, which adversely affected the patient's height and body weight. Regardless of the improvement in diarrhea, *H pylori* eradication itself may contribute to improvement in growth disorders. We believe that *H pylori* infection must be considered as a cause of chronic diarrhea and growth disorders even in early childhood.

In conclusion, *H pylori* infection can cause atrophic gastritis and chronic diarrhea without protein leakage as well as growth failure even in early infancy. We believe that early *H pylori* eradication therapy must be promoted for prevention of gastric cancer and improvement in growth disorders.

## Acknowledgments

The authors would like to thank the patient and his parents for consenting and allowing us to write and publish this case report.

## Author contributions

**Conceptualization:** Toshihiko Kakiuchi.

**Data curation:** Toshihiko Kakiuchi, Aiko Nakayama.

**Formal analysis:** Toshihiko Kakiuchi.

**Investigation:** Toshihiko Kakiuchi, Aiko Nakayama, Ryo Shimoda, Muneaki Matsuo.

**Methodology:** Toshihiko Kakiuchi.

**Project administration:** Muneaki Matsuo.

**Supervision:** Muneaki Matsuo.

**Validation:** Toshihiko Kakiuchi.

**Writing – original draft:** Toshihiko Kakiuchi, Aiko Nakayama.

**Writing – review and editing:** Muneaki Matsuo.

Toshihiko kakiuchi orcid: 0000-0002-9995-5522.
